# Supplementary material for: Structural mechanism of Necrocide 1 activation of human TRPM4 that triggers necrosis by sodium overload
Source: Nat Commun. 2026 Jun 24;17:7939. doi: 10.1038/s41467-026-74814-2 (PMC13448252; doi:10.1038/s41467-026-74814-2)
Supplement: Supplementary file 3 [file 41467_2026_74814_MOESM3_ESM.pdf]

nature portfolio

Peer Review File

Structural mechanism of Necrocide 1 activation of human TRPM4 that triggers necrosis by sodium overload

Corresponding Author: Professor Youxing Jiang

This file contains all reviewer reports in order by version, followed by all author rebuttals in order by version.

Version 0:

Reviewer comments:

Reviewer #1

(Remarks to the Author)
This manuscript reports a comprehensive structural and functional analysis of Necrocide 1 (NC1), a small molecule that selectively activates human TRPM4 to induce necrotic cell death via sodium overload. Using high-resolution cryo-EM structures, electrophysiology, mutagenesis, and cell death assays, the authors investigate NC1's binding site, mechanism of activation, PI(4,5)P_2 dependence, and the molecular basis of its species specificity. The work is technically strong. Since there is already a published structure of the closed and open structures of TRPM4 with an entirely unrelated molecule, decavanadate plus calcium, binding and opening the TRPM4 sodium channel at a physiologic temperature (Hu J, Park SJ, Walter T, Orozco IJ, O'Dea G, Ye X, Du J, Lü W. Nature. 2024 Jun;630(8016):509-515), there is a higher bar for this work to attain broad impact. A key unresolved issue is whether elevated intracellular calcium works together with NC1, or NC1 alone, is responsible for opening the TRPM4 channel. Also, the authors do not say much about the impact of the depletion of the TRPM4 inhibitor ATP on sustaining the open TRPM4 channel. The loss of energy through NC1-mediated mitochondrial dysfunction is nicely covered in their recent paper in this journal. Now that this paper is published its findings should be integrated into this manuscript.
Major points
1. A major issue is whether NC1 alone completely replaces calcium and opens the channel, or the initial opening of the TRPM4 channel involves both calcium and NC1. Ghosh et al., using a compound that is in the same general family as NC1, reported that their compound induced a very rapid increase in intracellular calcium in TRPM4 knockout breast cancer cells (Cancer Res. 2023 Sep 15;83(18):3115-3130). This type of rapid increase in intracellular calcium would probably impact opening the TRPM4 channel. The investigators have TRPM4 knockout breast cancer cells and should test whether NC1 causes a rapid increase in intracellular calcium that might contribute to opening the channel in this native model for TRPM4 action. Also, as discussed in (5) below, there is calcium density, consistent with bound calcium, in the NC1/PI(4,5)P_2-bound TRPM4 structure.
2. Opening the TRPM4 channel causes rapid sodium overload that causes mitochondrial dysfunction leading to loss of ATP. This topic was ably explored in this teams recent paper. Does this loss of ATP, which inhibits the TRPM4 channel, make a significant contribution to sustaining opening of the TRPM4 channel.
3. Decavanadate plus calcium binding and opening the closed TRPM4 sodium channel at physiologic temperature (Hu J, et al., Nature. 2024 630(8016):509-515) led to solved cryo-EM structures of TRPM4 in the closed state and open when bound to decavanadate plus calcium. Decavanadate is entirely different than NC1. The authors should do more to compare and contrast the open structure they see when NC1 is bound to TRPM4 to the published structure when decavanadate plus calcium is bound. The TRPM4 structure paper by Hu, J et al in Nature does not even appear to be cited in the references.
Minor Points
1. In Fig. 1a, intracellular Ca^{2+} is chelated using 1 mM EGTA to demonstrate Ca^{2+}-independent activation by NC1. There is a real question of whether EGTA effectively enters cells with intact plasma membranes and many researchers use a cell permeable calcium chelator, BAPTA-AM. This might help clarify the important question raised above on the potential role of increased calcium in opening the TRPM4 channel in the presence of NC1.
2. Have the authors examined, structurally or functionally, whether simultaneous presence of NC1 and Ca^{2+} produces additive or distinct conformational effects on channel gating, compared to the presence of NC1 alone?
3. In Fig. 4a and 4b, desensitization following Ca^{2+} activation is attributed to PI(4,5)P_2 depletion via PLC activation. While consistent with prior literature, it would strengthen the interpretation if the authors could clarify whether PLC inhibition (e.g., U73122) affects the observed desensitization under their experimental conditions, or explicitly state this as an assumption based on established mechanisms.

4. Does prolonged NC1 exposure alone induce any form of channel desensitization or inactivation, analogous to Ca^{2+}-induced desensitization? Clarification of this point would improve understanding of NC1 gating kinetics.
5. In Fig. 5b, Ca^{2+} density is observed in the NC1/PI(4,5)P_2-bound TRPM4 structure despite no exogenous Ca^{2+} being added. While the EGTA-controlled structure is reassuring, additional clarification of residual Ca^{2+} levels during purification and data collection would help readers better assess whether NC1 activates TRPM4 independently of Ca^{2+} rather than working together with Ca^{2+}, or sensitizing the channel to trace Ca^{2+}.
6. The functional relevance of the species-specific effects is convincingly demonstrated using HeLa cells expressing human or mouse TRPM4 variants. The authors may wish to test the same system in an endogenous mouse cancer cell line, which could further enhance the physiological relevance of this finding.
This manuscript contains important studies that explain the difference in activation between mouse and human TRPM4 and other high quality experimental work. Addressing the several issues raised would materially strengthen this impressive manuscript.

Reviewer #2

(Remarks to the Author)
I co-reviewed this manuscript with one of the reviewers who provided the listed reports. This is part of the Nature Communications initiative to facilitate training in peer review and to provide appropriate recognition for Early Career Researchers who co-review manuscripts.

Reviewer #3

(Remarks to the Author)
This study, entitled "Structural mechanism of Necrocide 1 activation of human TRPM4 that triggers necrosis by sodium overload," investigates the molecular mechanism of human TRPM4 channel activation via Necrocide (NC1) and its species-specificity using cryo-EM approaches and electrophysiological experiments. The author found that NC1 binds to a pocket in human TRPM4 near the well-described calcium site, thereby allowing channel opening. In addition, they showed that membrane PI(4,5)P2 is required to stabilize the open state of the human TRPM4 channel induced by interaction between the channel and NC1. Moreover, they demonstrate species-specificity by showing that this feature is not due to decreased binding between NC1 and the mouse TRPM4 channel, but rather to species-specific conformational changes that destabilize the selectivity filter and inactivate the mouse TRPM4 channel. Finally, they identify three key residues in the mouse TRPM4 channel that render it Insensitive to NC1. Overall, the manuscript is solid and well presented. Nevertheless, I have a few comments.

Major comments:

Figures 4 and S4. Based on the point mutations of the PIP2 interaction pocket (R664, K925, and K928), you conclude that the PIP2 is required prior to the application of NC1 to induce its effect. However, based on the figures, I will be cautious in interpreting them because the expression at the cell surface of the two mutant hTRPM4 proteins, p.K925A and p.K928A, appears altered. If the expression at the plasma membrane of those variants is reduced compared to the wild-type channel, the increase in the current density after application of NC1 will also be lower (assuming that the efficiency of the NC1 is independent of the point mutation introduced). It is important in this case to quantify the NC1 effect in percentage. The best will be to have the TRPM4 current recorded under the following conditions:
1) Without calcium
2) With calcium
3) Without calcium
4) Without calcium + 10 µM NC1.

Under these conditions, the first two steps allow quantification of the current calcium-activated, and the last step allows you to estimate, as a percentage, the effect of NC1 when compared to the calcium-activated one.

Figure 6c. Similarly to the above-mentioned comment. If you really want to present the bar graph suggesting that the triple mutant mTRPM4 behaves like the human TRPM4 after application of NC1, you should present it as a percentage. However, in my view, the key message from these experiments is that the triple mouse TRPM4 mutant is sensitive to NC1. In this case, I will not present the bar graph and instead replace it with a table showing (-) (not sensitive) and (+) (sensitive), without any quantification.

Minor comments:

In general, it is more appropriate to present dot plots rather than bar graphs.

Please check when a figure is at the beginning of a sentence. In this case, the figure should be written in full letters.

Please include stars on figures when significance is reached (e.g., in figures 2d and 2e, or in figures 4d and 4e).

Reviewer #4

(Remarks to the Author)
Review of “Structural mechanism of Necrocide 1 activation of human TRPM4 that triggers necrosis by sodium overload” by Teixeira-Duarte et al.

In this manuscript, Teixeira-Duarte et al. investigate the structural basis of TRPM4 activation by the small molecule Necrocide 1 (NC1). The authors demonstrate that NC1 activates transiently expressed human TRPM4 in HeLa cells, leading to cell death. The cell death assay established in this study provides an effective functional complement to the structural analysis.

The authors present multiple high-quality cryo-EM structures of NC1- and PIP2-bound human and mouse TRPM4. In addition, they determine structures of a mutant mouse TRPM4 variant and of the channel in the presence of the drug and a chelator. Together, these data provide mechanistic insight into the species specificity of NC1 and offer a plausible structural explanation for why NC1 activates human but not mouse TRPM4.

Overall, the study is carefully conducted and technically strong. The integration of high-resolution cryo-EM, electrophysiology, and functional cell death assays provides converging lines of evidence supporting the proposed mechanism. While some aspects of the interpretation would benefit from minor clarification (see below), the central conclusions are well supported by the data.

The manuscript represents a substantial contribution to the understanding of TRPM4 pharmacology and channel activation mechanisms and will be of broad interest to the ion channel and structural biology communities. I support its publication in Nature Communications following minor revision.

Minor comment:

I recommend that the authors provide map density for the residues surrounding the drug-binding site at the same contour threshold used for the ligand. This should be done for the all the structures presented similar to what is shown in Fig S5b. This would allow readers to more directly assess the quality of the density and the robustness of the modeled drug–protein interactions.

Version 1:

Reviewer comments:

Reviewer #1

(Remarks to the Author)
The revised manuscript has responded in a satisfactory way to most suggestions in the review.

Enthusiasm is somewhat muted because the authors did not do one relatively simple experiment that was proposed: Putting the human TRPM4 into a mouse cell to see if that is sufficient to make mouse breast cancer cells sensitive to the compound. Their written response to this comment is a bit awkward.

Reviewer #2

(Remarks to the Author)
I co-reviewed this manuscript with one of the reviewers who provided the listed reports. This is part of the Nature Communications initiative to facilitate training in peer review and to provide appropriate recognition for Early Career Researchers who co-review manuscripts.

Reviewer #3

(Remarks to the Author)
Thanks to the authors for the clarification and the added experiments.
Overall, this manuscript is convincing, and I have no other comments.

Reviewer #4

(Remarks to the Author)
I have no further concerns and support the publication of this work in Nature Communications.

Version 2:

Reviewer comments:

Reviewer #1

(Remarks to the Author)
The authors inserted human TRPm4 into mouse cell lines and show that at the high dose of 1 uM the compound kills most of the mouse cells.

This responds adequately to the comment.

Open Access This Peer Review File is licensed under a Creative Commons Attribution 4.0 International License, which permits use, sharing, adaptation, distribution and reproduction in any medium or format, as long as you give appropriate credit to the original author(s) and the source, provide a link to the Creative Commons license, and indicate if changes were made.
In cases where reviewers are anonymous, credit should be given to 'Anonymous Referee' and the source.
The images or other third party material in this Peer Review File are included in the article's Creative Commons license, unless indicated otherwise in a credit line to the material. If material is not included in the article's Creative Commons license and your intended use is not permitted by statutory regulation or exceeds the permitted use, you will need to obtain permission directly from the copyright holder.
To view a copy of this license, visit https://creativecommons.org/licenses/by/4.0/

Response to Reviewers

Reviewer #1:

This manuscript reports a comprehensive structural and functional analysis of Necrocide 1 (NC1), a small molecule that selectively activates human TRPM4 to induce necrotic cell death via sodium overload. Using high-resolution cryo-EM structures, electrophysiology, mutagenesis, and cell death assays, the authors investigate NC1's binding site, mechanism of activation, PI(4,5)P_2 dependence, and the molecular basis of its species specificity. The work is technically strong. Since there is already a published structure of the closed and open structures of TRPM4 with an entirely unrelated molecule, decavanadate plus calcium, binding and opening the TRPM4 sodium channel at a physiologic temperature (Hu J, Park SJ, Walter T, Orozco IJ, O'Dea G, Ye X, Du J, Lü W. Nature. 2024 Jun;630(8016):509-515), there is a higher bar for this work to attain broad impact. A key unresolved issue is whether elevated intracellular calcium works together with NC1, or NC1 alone, is responsible for opening the TRPM4 channel. Also, the authors do not say much about the impact of the depletion of the TRPM4 inhibitor ATP on sustaining the open TRPM4 channel. The loss of energy through NC1-mediated mitochondrial dysfunction is nicely covered in their recent paper in this journal. Now that this paper is published its findings should be integrated into this manuscript.

We appreciate reviewer #1's positive comments and constructive suggestions. The general comments raised by the reviewer are addressed as follows:

As described in the electrophysiology experiments (whole-cell recordings) shown in Figure 1, EGTA was included in the pipette solution (cytosolic side), which effectively buffers intracellular Ca^{2+}. This eliminates the potential contribution of cytosolic Ca^{2+} to channel activation and confirms that NC1 activation of TRPM4 is Ca^{2+}-independent. In fact, all recordings of NC1 activation of hTRPM4 presented in this study were performed under Ca^{2+}-free conditions (in the presence of EGTA). A similar result was also discussed in our recent study that defines the role of TRPM4 in NC1-induced necrosis (PMID: 39915626).

The current study focuses on the pharmacology of TRPM4, specifically the structural mechanism underlying NC1 activation of human TRPM4 and its species specificity. The mechanism of ATP-dependent inhibition, which has been extensively discussed in our previous studies (PMID: 29211714 and PMID: 41174278), is beyond the main scope of the present work. It is worth noting that TRPM4 is inhibited by free ATP (ATP^{4-}) but not by Mg-chelated ATP (Mg-ATP). Under physiological conditions, where the concentration of free cytosolic ATP is low, NC1 induces robust activation of hTRPM4, and we did not observe obvious ATP-mediated inhibition in our recordings. Because ATP depletion is a consequence of NC1-induced NECSO, we do not expect this depletion to play an additional role in sustaining NC1-induced TRPM4 activation.

Major points

1. A major issue is whether NC1 alone completely replaces calcium and opens the channel, or the initial opening of the TRPM4 channel involves both calcium and NC1. Ghosh et al., using a compound that is in the same general family as NC1, reported that their compound induced a very rapid increase in intracellular calcium in TRPM4 knockout breast cancer cells (Cancer Res. 2023 Sep 15;83(18):3115-3130). This type of rapid increase in intracellular calcium would probably impact opening the TRPM4 channel. The investigators have TRPM4 knockout breast cancer cells and should test whether NC1 causes a rapid increase in intracellular calcium that might contribute to opening the channel in this native model for TRPM4 action. Also, as discussed in (5) below, there is calcium density, consistent with bound calcium, in the NC1/PI(4,5)P_2-bound TRPM4 structure.

To address this question, we monitored cytosolic calcium in MCF7 cells using both the chemical calcium indicator Fluo-4 AM and the genetically encoded calcium indicator R-GECO1. We observed an NC1-induced increase in cytosolic Ca^{2+} in a TRPM4-dependent manner (Figure 1 in this response). However, in contrast to the findings of Ghosh et al., this NC1-induced Ca^{2+} elevation develops slowly and can be abolished either by TRPM4 knockout or by depletion of extracellular sodium (Figure 1). These results suggest that the observed Ca^{2+} increase is a consequence of TRPM4 activation rather than a prerequisite for channel opening. Specifically, sodium influx through TRPM4 and the resulting membrane depolarization appear to be required for the subsequent rise in cytosolic Ca^{2+}. This interpretation is consistent with a model in which TRPM4-mediated Na^+ entry depolarizes the membrane, thereby facilitating secondary Ca^{2+} influx, likely through voltage-dependent calcium channels or altered ion exchanger activity.

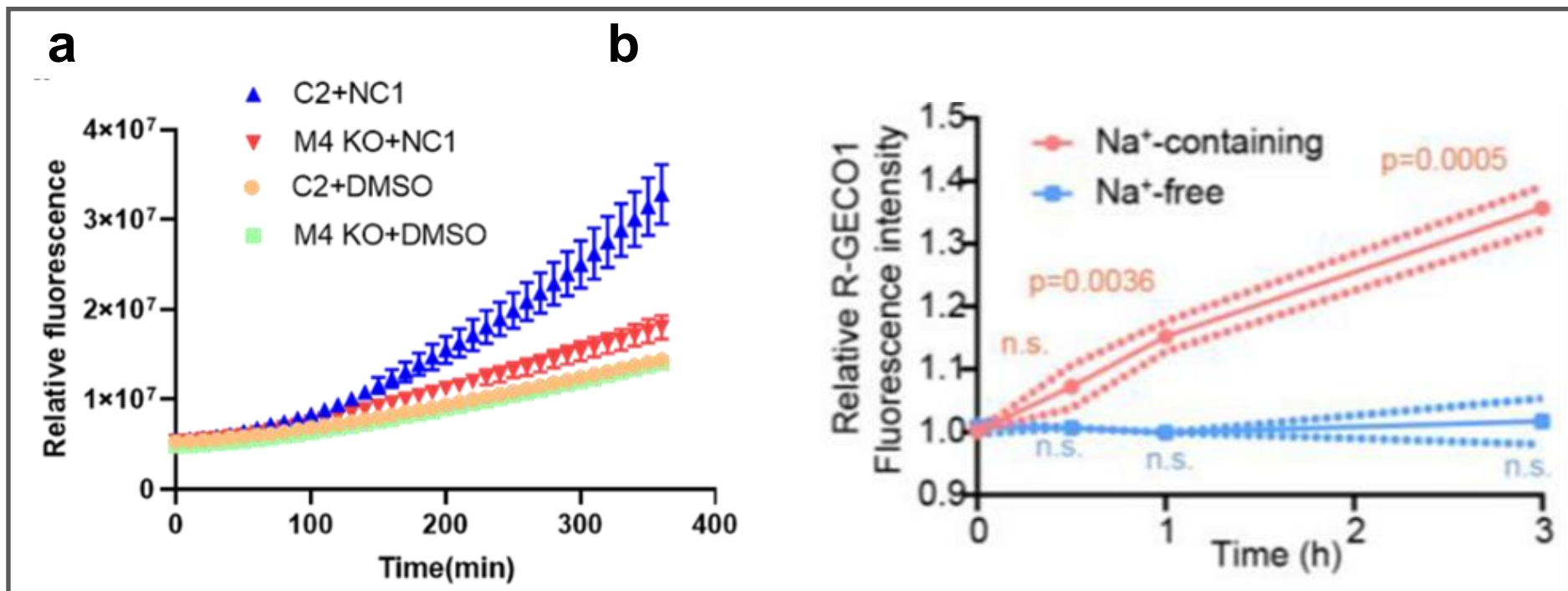

Figure 1. NC1 induces cytosolic calcium elevation in a hTRPM4- and sodium-dependent manner. (a) MCF7 control (C2) and TRPM4-knockout (M4 KO) cells preloaded with Fluo-4 AM were treated with DMSO or 100 nM NC1. Time-lapse fluorescence intensity was recorded using a microplate reader to monitor cytosolic calcium levels. (b) Quantification of R-GECO1 fluorescence signals. For each condition, fluorescence intensity was normalized to the respective untreated control (0 h). Statistical analysis was performed using one-way ANOVA. Data are presented as mean ± SEM.

In addition, the functional assays presented in this study demonstrate that NC1 activates TRPM4 in a Ca^{2+}-independent manner, and increases in cytosolic Ca^{2+} do not potentiate NC1-mediated activation (Figure 2 in this response and also included in the revised manuscript as Supplementary Figure 5c). Similar conclusions were also discussed in our recent study (PMID: 39915626).

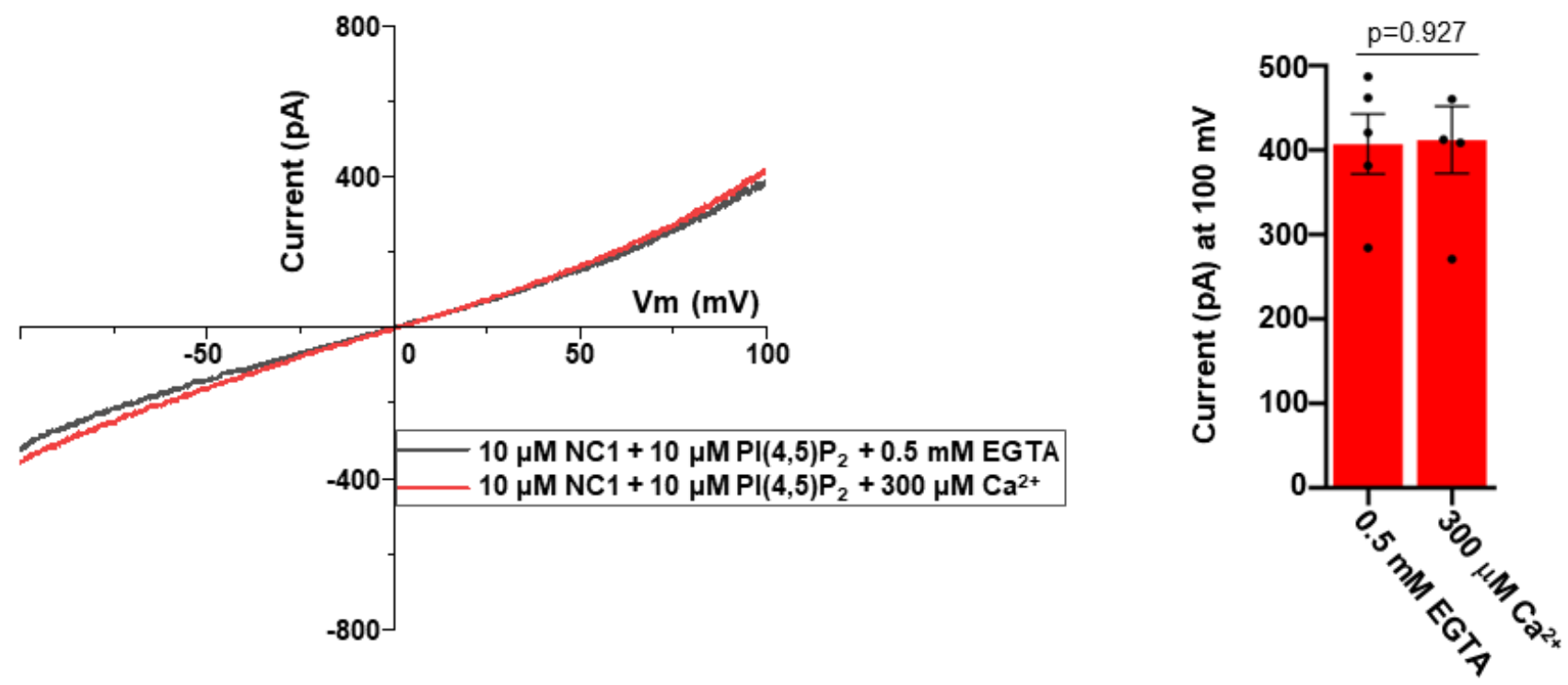

Figure 2: Sample I-V curves from an inside-out patch of NC1/PI(4,5)P_2-activated hTRPM4 with 10 μM PI(4,5)P_2 diC8 and 10 μM NC1 in the bath (cytosolic) in the absence (with EGTA) or presence of Ca^{2+}. The bar graph on the right displays the outward currents recorded at +100 mV in the inside-out patches with 10 μM NC1, 10 μM PI(4,5)P_2 di-C8, and either 0.5 mM EGTA or 300 μM Ca^{2+} in the bath (cytosolic). Bars represent mean ± SEM of n=5 independent replicates (shown as dots). p-value was calculated using a two-sided Student's t-test.

The NC1 binding site does not overlap with the Ca^{2+} binding site, and we indeed observed Ca^{2+} density in the NC1/PI(4,5)P_2-bound open TRPM4 structure obtained under nominally Ca^{2+}-free conditions. To further confirm the Ca^{2+} independence of NC1 activation, we also determined the NC1/PI(4,5)P_2-bound structure in the presence of EGTA (Supplementary Figure 5a&b in the manuscript). In this structure, no Ca^{2+} density is observed, and the channel still adopts the same open conformation as in the Ca^{2+}-bound structure. This result has been discussed in the manuscript.

2. <u>Opening the TRPM4 channel causes rapid sodium overload that causes mitochondrial dysfunction leading to loss of ATP. This topic was ably explored in this teams recent paper. Does this loss of ATP, which inhibits the TRPM4 channel, make a significant contribution to sustaining opening of the TRPM4 channel.</u>

As discussed in our response to the reviewer's general comments, TRPM4 is inhibited by free ATP (ATP^{4-}) but not by Mg-chelated ATP (Mg-ATP). Under physiological conditions, where the concentration of free cytosolic ATP is low, NC1 induces robust activation of hTRPM4, and we did not observe obvious ATP-mediated inhibition in our recordings. Because ATP depletion is a consequence of NC1-induced NECSO, we do not expect this depletion to play an additional role in sustaining NC1-induced TRPM4 activation.

3. Decavanadate plus calcium binding and opening the closed TRPM4 sodium channel at physiologic temperature (Hu J, et al., Nature. 2024 630(8016):509-515) led to solved cryo-EM structures of TRPM4 in the closed state and open when bound to decavanadate plus calcium. Decavanadate is entirely different than NC1. The authors should do more to compare and contrast the open structure they see when NC1 is bound to TRPM4 to the published structure when decavanadate plus calcium is bound. The TRPM4 structure paper by Hu, J et al in Nature does not even appear to be cited in the references.

The study by Lü's group on TRPM4 activation by decavanadate/Ca^{2+} at physiological temperature has been extensively discussed and compared in our recent paper examining TRPM4 activation by PI(4,5)P_2/Ca^{2+} and its inactivation by ATP (PMID: 41174278). In that study, we demonstrated that decavanadate mimics the effect of PI(4,5)P_2 in promoting TRPM4 activation, and that the open structures obtained with PI(4,5)P_2/Ca^{2+} and decavanadate/Ca^{2+} are highly similar. The work of Lü's group has been cited in the revised manuscript.

Minor Points

1. In Fig. 1a, intracellular Ca^{2+} is chelated using 1 mM EGTA to demonstrate Ca^{2+}-independent activation by NC1. There is a real question of whether EGTA effectively enters cells with intact plasma membranes and many researchers use a cell permeable calcium chelator, BAPTA-AM. This might help clarify the important question raised above on the potential role of increased calcium in opening the TRPM4 channel in the presence of NC1.

The recording shown in Figure 1a was performed using the whole-cell patch-clamp configuration with EGTA included in the pipette solution, which is equivalent to introducing EGTA directly into the cytosol to buffer intracellular Ca^{2+}.

2. Have the authors examined, structurally or functionally, whether simultaneous presence of NC1 and Ca^{2+} produces additive or distinct conformational effects on channel gating, compared to the presence of NC1 alone?

We did measure TRPM4 activation in the presence of both NC1 and Ca^{2+}. Following NC1 activation, the addition of Ca^{2+} did not produce any additional effect (Figure 2 in response to reviewer's 1st major point). This result is also included in the revised manuscript as Supplementary Figure 5c. A similar observation was also reported in our recent study (PMID: 39915626).

For the structural analysis, we present two structures in this study: one NC1/PI(4,5)P_2–activated structure with bound Ca^{2+} (Figure 5a&b in the main text), which was obtained under nominal Ca^{2+}-free conditions, and another NC1/PI(4,5)P_2-activated structure determined in the absence of Ca^{2+} (in the presence of EGTA) (Supplementary Figure. 5a&b). These two structures are virtually identical, confirming that Ca^{2+} does not contribute to NC1-mediated activation of TRPM4.

3. In Fig. 4a and 4b, desensitization following Ca^{2+} activation is attributed to $PI(4,5)P_2$ depletion via PLC activation. While consistent with prior literature, it would strengthen the interpretation if the authors could clarify whether PLC inhibition (e.g., U73122) affects the observed desensitization under their experimental conditions, or explicitly state this as an assumption based on established mechanisms.

The exact experiment proposed by the reviewer has, in fact, been performed in a previous study investigating TRPM4 desensitization caused by Ca^{2+}-induced phospholipase C activation (PMID:16424899), which is cited in our manuscript.

4. Does prolonged NC1 exposure alone induce any form of channel desensitization or inactivation, analogous to Ca^{2+}-induced desensitization? Clarification of this point would improve understanding of NC1 gating kinetics.

Under our experimental conditions, with recordings extending up to 10 minutes after NC1 activation, we did not observe any detectable channel desensitization.

5. In Fig. 5b, Ca^{2+} density is observed in the NC1/$PI(4,5)P_2$-bound TRPM4 structure despite no exogenous Ca^{2+} being added. While the EGTA-controlled structure is reassuring, additional clarification of residual Ca^{2+} levels during purification and data collection would help readers better assess whether NC1 activates TRPM4 independently of Ca^{2+} rather than working together with Ca^{2+}, or sensitizing the channel to trace Ca^{2+}.

As discussed in our previous responses, we have functionally demonstrated that NC1 activation of TRPM4 is Ca^{2+}-independent. In addition, we present two structures in this study: one NC1/$PI(4,5)P_2$–activated structure with bound Ca^{2+} (Figure 5a&b) and another NC1/$PI(4,5)P_2$–activated structure determined in the absence of Ca^{2+} (in the presence of EGTA) (Supplementary Figure. 5a&b). These two structures are virtually identical, confirming that although Ca^{2+} binding does not compete with NC1, it does not contribute to NC1-mediated activation of TRPM4.

6. The functional relevance of the species-specific effects is convincingly demonstrated using HeLa cells expressing human or mouse TRPM4 variants. The authors may wish to test the same system in an endogenous mouse cancer cell line, which could further enhance the physiological relevance of this finding.

The experiment suggested by the reviewer was performed in our earlier study that defined NC1 activation of human TRPM4, which induces NECSO, and demonstrated its specificity for human TRPM4 (PMID: 39915626). In that study, we examined NC1-induced cell death across multiple human cancer cell lines and showed that these cells exhibit hTRPM4 expression–

dependent sensitivity to NC1. We also evaluated NC1 sensitivity in normal and cancer cell lines derived from mouse and hamster. These cells were found to be over 1,000-fold more resistant to NC1 than human cells, despite some mouse cell lines expressing TRPM4 at levels comparable to several human cell lines. These findings suggest that species-specific differences in TRPM4 underlie the observed resistance to NC1.

Reviewer #2:

I co-reviewed this manuscript with one of the reviewers who provided the listed reports. This is part of the Nature Communications initiative to facilitate training in peer review and to provide appropriate recognition for Early Career Researchers who co-review manuscripts.

Reviewer #2 co-reviewed the manuscript.

Reviewer #3:

This study, entitled "Structural mechanism of Necrocide 1 activation of human TRPM4 that triggers necrosis by sodium overload," investigates the molecular mechanism of human TRPM4 channel activation via Necrocide (NC1) and its species-specificity using cryo-EM approaches and electrophysiological experiments. The author found that NC1 binds to a pocket in human TRPM4 near the well-described calcium site, thereby allowing channel opening. In addition, they showed that membrane PI(4,5)P2 is required to stabilize the open state of the human TRPM4 channel induced by interaction between the channel and NC1. Moreover, they demonstrate species-specificity by showing that this feature is not due to decreased binding between NC1 and the mouse TRPM4 channel, but rather to species-specific conformational changes that destabilize the selectivity filter and inactivate the mouse TRPM4 channel. Finally, they identify three key residues in the mouse TRPM4 channel that render it Insensitive to NC1. Overall, the manuscript is solid and well presented. Nevertheless, I have a few comments.

We appreciate reviewer #3's positive comments and constructive suggestions. The points raised by the reviewer are addressed as follows:

Major comments:

Figures 4 and S4. Based on the point mutations of the PIP2 interaction pocket (R664, K925, and K928), you conclude that the PIP2 is required prior to the application of NC1 to induce its effect. However, based on the figures, I will be cautious in interpreting them because the expression at the cell surface of the two mutant hTRPM4 proteins, p.K925A and p.K928A, appears altered. If the expression at the plasma membrane of those variants is reduced compared to the wild-type channel, the increase in the current density after application of NC1 will also be lower (assuming that the efficiency of the NC1 is independent of the point mutation introduced). It is important in this case to quantify the NC1 effect in percentage. The best will be to have the TRPM4 current recorded under the following conditions:

1) Without calcium
2) With calcium
3) Without calcium
4) Without calcium + 10 μM NC1.

Under these conditions, the first two steps allow quantification of the current calcium-activated, and the last step allows you to estimate, as a percentage, the effect of NC1 when compared to the calcium-activated one.

We agree with the reviewer that differences in protein expression levels could potentially contribute to the variation in currents observed for the PIP2-site mutants. Because PIP2 is essential for both NC1- and Ca^{2+}-mediated activation, mutations at the PIP2 binding site would be expected to exert a similar diminishing effect on TRPM4 currents activated by either NC1 or Ca^{2+}. Consistent with this expectation, in our recent study on PIP2/Ca^{2+} activation (PMID: 41174278), we observed that the same PIP2-site mutations markedly reduce Ca^{2+}-activated channel currents, similar to their effect on NC1 activation. Therefore, the experiment suggested by the reviewer - normalizing NC1-induced currents to Ca^{2+}-activated currents - would not allow us to quantitatively assess the mutation effects.

To minimize the potential influence of protein expression variability on channel currents, we employed two complementary approaches. First, because all TRPM4 constructs used for electrophysiological recordings were EGFP-tagged, we selected cells with comparable fluorescence intensity for the recordings to reduce variability arising from differences in expression levels. Second, we performed a surface protein biotinylation and pull-down assay in the same HEK293 cells used for electrophysiology. The results show that the surface expression levels of these mutants are comparable to those of the WT channel. These data have been included as Supplementary Figure 4a in the revised manuscript.

Figure 6c. Similarly to the above-mentioned comment. If you really want to present the bar graph suggesting that the triple mutant mTRPM4 behaves like the human TRPM4 after application of NC1, you should present it as a percentage. However, in my view, the key message from these experiments is that the triple mouse TRPM4 mutant is sensitive to NC1. In this case, I will not present the bar graph and instead replace it with a table showing (-) (not sensitive) and (+) (sensitive), without any quantification.

Because some mutants appear to partially restore NC1 sensitivity in mTRPM4, we felt it was important to provide quantitative measurements rather than a binary representation of + (sensitive) or − (insensitive). As these mutations do not target the Ca^{2+}- or PIP2-binding sites, they are not expected to affect Ca^{2+}/PIP2-mediated activation of the channel. Following the reviewer's previous suggestion, we performed control experiments measuring Ca^{2+}/PIP2-activated currents for all of these mutants using inside-out patch recordings, and we also compared their sensitivity to NC1/PIP2 activation. The results show that all mutants produced Ca^{2+}/PIP2-elicited currents comparable to those of wild-type mTRPM4, indicating similar channel expression levels between the mutants and the WT. However, only mutants containing the S1060R mutation could be partially (single and double mutants) or fully (triple mutant) activated by NC1/PIP_2, consistent with the observations from the whole-cell recordings. These data have been included in the revised manuscript as Supplementary Figure 8a&b).

Minor comments:

In general, it is more appropriate to present dot plots rather than bar graphs.

We have incorporated dot plots into the bar graphs as suggested in the revised manuscript.

Please check when a figure is at the beginning of a sentence. In this case, the figure should be written in full letters.

Corrected as suggested.

Please include stars on figures when significance is reached (e.g., in figures 2d and 2e, or in figures 4d and 4e).

Statistical significance, represented by asterisks, has been included in the relevant figures in the revised manuscript as suggested.

Reviewer #4:

Review of “Structural mechanism of Necrocide 1 activation of human TRPM4 that triggers necrosis by sodium overload” by Teixeira-Duarte et al.

In this manuscript, Teixeira-Duarte et al. investigate the structural basis of TRPM4 activation by the small molecule Necrocide 1 (NC1). The authors demonstrate that NC1 activates transiently expressed human TRPM4 in HeLa cells, leading to cell death. The cell death assay established in this study provides an effective functional complement to the structural analysis.

The authors present multiple high-quality cryo-EM structures of NC1- and PIP2-bound human and mouse TRPM4. In addition, they determine structures of a mutant mouse TRPM4 variant and of the channel in the presence of the drug and a chelator. Together, these data provide mechanistic insight into the species specificity of NC1 and offer a plausible structural explanation for why NC1 activates human but not mouse TRPM4.

Overall, the study is carefully conducted and technically strong. The integration of high-resolution cryo-EM, electrophysiology, and functional cell death assays provides converging lines of evidence supporting the proposed mechanism. While some aspects of the interpretation would benefit from minor clarification (see below), the central conclusions are well supported by the data.

The manuscript represents a substantial contribution to the understanding of TRPM4 pharmacology and channel activation mechanisms and will be of broad interest to the ion channel and structural biology communities. I support its publication in Nature Communications following minor revision.

We appreciate Review #4’s positive critiques of our manuscript. The minor point raised by the reviewer is addressed as follows:

Minor comment:

I recommend that the authors provide map density for the residues surrounding the drug-binding site at the same contour threshold used for the ligand. This should be done for the all the structures presented similar to what is shown in Fig S5b. This would allow readers to more directly assess the quality of the density and the robustness of the modeled drug–protein interactions.

We have modified the figures as suggested in the revised manuscript.

Response to Reviewers

Reviewer #1:

The revised manuscript has responded in a satisfactory way to most suggestions in the review. Enthusiasm is somewhat muted because the authors did not do one relatively simple experiment that was proposed: Putting the human TRPM4 into a mouse cell to see if that is sufficient to make mouse breast cancer cells sensitive to the compound. Their written response to this comment is a bit awkward.

As proposed, we expressed human TRPM4 (hTRPM4) in two mouse breast cancer cell lines to evaluate their sensitivity to NC1. Vector-transfected control cells remained resistant to NC1, whereas hTRPM4 expression markedly increased sensitivity, as evidenced by dose-dependent cell death (Figure 1a-d in this response and also included in the revised manuscript as Supplementary Fig. 11). These results indicate that hTRPM4 is sufficient to confer NC1 sensitivity in otherwise resistant mouse cancer cells.

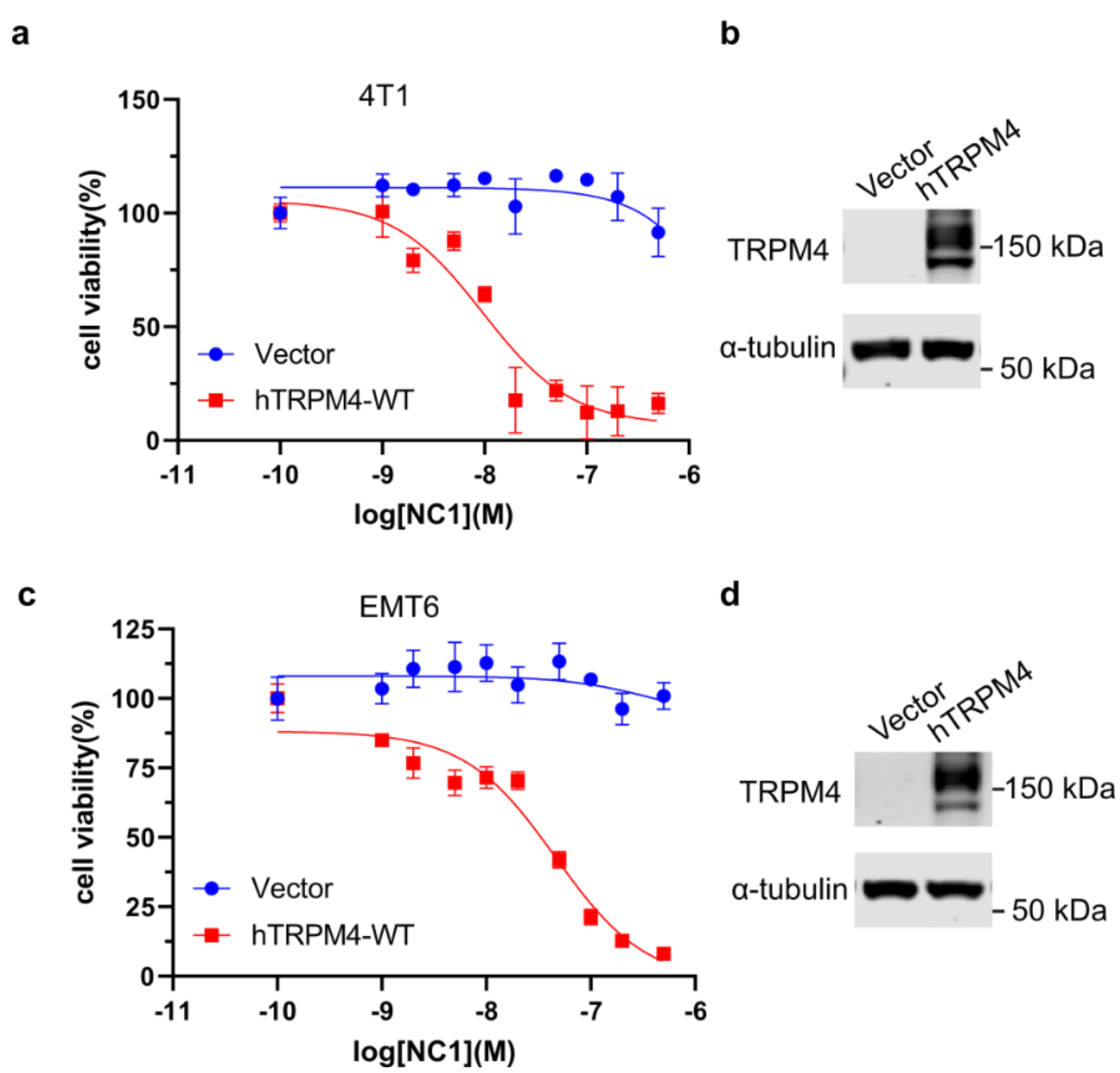

Figure 1. Human TRPM4 expression sensitizes mouse cancer cells to NC1.

NC1-induced cytotoxicity was assessed in 4T1 (a,b) and EMT6 (c,d) cells transfected with either vector control or human TRPM4. Cell viability data are presented as mean ± s.d. (n = 3 independent replicates).

Reviewer #2:

I co-reviewed this manuscript with one of the reviewers who provided the listed reports. This is part of the Nature Communications initiative to facilitate training in peer review and to provide appropriate recognition for Early Career Researchers who co-review manuscripts.

Reviewer #3:

Thanks to the authors for the clarification and the added experiments.
Overall, this manuscript is convincing, and I have no other comments.

This reviewer's concerns have been satisfactorily addressed in the previous revision

Reviewer #4 (Remarks to the Author):

I have no further concerns and support the publication of this work in Nature Communications.

This reviewer's concerns have been satisfactorily addressed in the previous revision

Response to Reviewers

Reviewer #1:

The authors inserted human TRPm4 into mouse cell lines and show that at the high dose of 1 uM the compound kills most of the mouse cells.

This responds adequately to the comment.

We appreciate the support from this reviewer.

Reviewer #2:

I co-reviewed this manuscript with one of the reviewers who provided the listed reports. This is part of the Nature Communications initiative to facilitate training in peer review and to provide appropriate recognition for Early Career Researchers who co-review manuscripts.

Reviewer #3:

No additional comment from this reviewer in this revision round.

We appreciate the support from this reviewer.

Reviewer #4 (Remarks to the Author):

No additional comment from this reviewer in this revision round.

We appreciate the support from this reviewer.
